# Cardiac involvement in systemic sclerosis: mechanisms, manifestations and management

**DOI:** 10.1093/rheumatology/keag064

**Published:** 2026-02-04

**Authors:** Chin Yit Soo, Nicholas Jex, Lesley-Anne Bissell, Francesco Del Galdo, Sven Plein

**Affiliations:** Biomedical Imaging Science Department, Leeds Institute of Cardiovascular and Metabolic Medicine, University of Leeds, United Kingdom; Biomedical Imaging Science Department, Leeds Institute of Cardiovascular and Metabolic Medicine, University of Leeds, United Kingdom; Leeds Institute of Rheumatic and Musculoskeletal Medicine, University of Leeds, United Kingdom; Leeds Institute of Rheumatic and Musculoskeletal Medicine, University of Leeds, United Kingdom; Biomedical Imaging Science Department, Leeds Institute of Cardiovascular and Metabolic Medicine, University of Leeds, United Kingdom

**Keywords:** systemic sclerosis, primary heart involvement, cardiac function, prognosis, cardiac magnetic resonance, coronary microvascular circulation, cardiac fibrosis, cardiac energetics

## Abstract

Cardiovascular disease remains a leading cause of mortality in systemic sclerosis (SSc). This review summarizes key mechanisms in the pathophysiology of SSc primary heart involvement (SSc-PHI), presents the main clinical manifestations and their management and discusses unmet needs in SSc-PHI with potential solutions. The pathophysiology of SSc-PHI involves both vascular and immune-mediated pathways, exacerbated by the NLRP3 inflammasome and pro-fibrotic cytokines, leading to myocardial fibrosis as the main cardiac manifestation. Regular screening is recommended to detect SSc-PHI, incorporating clinical assessments, cardiac biomarkers and transthoracic echocardiography. Cardiovascular magnetic resonance imaging (CMR) enables detection of subclinical cardiac involvement, with certain CMR phenotypes holding prognostic significance. Early identification of subclinical disease is a critical unmet need to enhance risk stratification and improve outcomes. Emerging programs and cardiology-rheumatology collaboration will enhance interdisciplinary care and diagnostic strategies.

Rheumatology key messages

- Primary heart involvement in systemic sclerosis is difficult to diagnose but has high mortality.
- Subclinical SSc-PHI can be assessed non-invasively on cardiac magnetic resonance imaging with certain prognostic information.
- Reliable diagnostic pathways support the development of targeted therapies for SSc-PHI (which are currently lacking).

## Introduction

SSc, or scleroderma, is a rare multi-system autoimmune disorder which causes small vessel vasculopathy, inflammation and progressive fibrosis of the skin and internal organs [1]. Its prevalence ranges from 3.1 to 144.5 per 100 000, influenced by ethnicity, standards in disease recognition and diagnosis, with approximately 5-fold higher prevalence in women [2–4]. SSc is associated with a significant excess mortality with a standardized mortality rate of 3.5 and cumulative survival from diagnosis of 74.9% at 5 years and 62.5% at 10 years in a meta-analysis [5]. Combined cardiac causes accounted for 31% and 27% mortality in a French and EUSTAR cohort, respectively [6]. In the EUSTAR cohort, 12% of deaths were due to systemic sclerosis primary heart involvement (SSc-PHI) alone [6].

SSc-PHI is defined as cardiac abnormalities that are predominantly attributable to SSc rather than other causes and/or complications [7, 8]. It can present as pericarditis, myocarditis, tissue fibrosis with conduction abnormalities, bi-ventricular diastolic and systolic dysfunction [9–12]. SSc-PHI by definition excludes heart involvement that arises secondary to other SSc organ manifestations (such as renal, pulmonary vascular or pulmonary parenchymal disease) or coincidental disease (i.e., ischemic heart disease) [8]. Although conceptually appealing, distinguishing primary from secondary cardiac involvement is challenging in practice, as symptoms and clinical manifestations overlap and non-cardiac involvement in SSc can mask the symptoms of primary and secondary cardiovascular disease. For example, the symptoms of pulmonary arterial hypertension (PAH) and/or interstitial lung disease (ILD) are difficult to distinguish from those of associated right ventricular (RV) systolic dysfunction or SSc-PHI and both can be associated with abnormal cardiac blood biomarkers and abnormalities on imaging tests [8, 13, 14].

The pathological hallmarks of SSc-PHI include microvascular ischaemia and myocardial inflammation, both contributing to perivascular and myocardial fibrosis with an increasing recognition of the role of accelerated macrovascular disease [15, 16]. In this review, we summarize the main mechanisms of SSc-PHI, discuss its most common manifestations and their management and give an overview of the unmet needs in the management of SSc-PHI with potential solutions.

## Mechanisms of SSc-PHI

**Figure 1:**
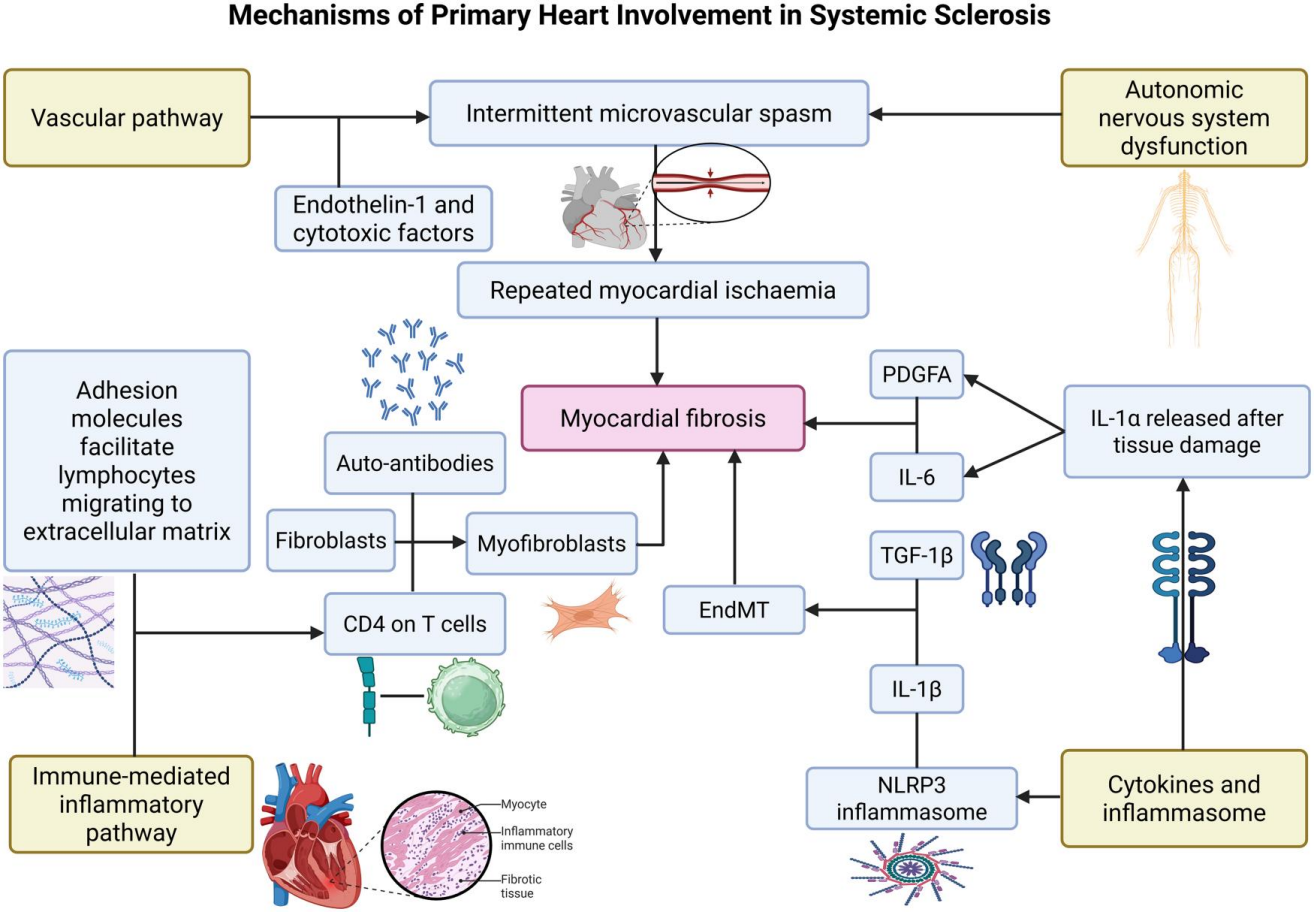
Mechanisms of primary heart involvement in systemic sclerosis. In vascular pathway, there is intermittent microvascular spasm, compounded by endothelin-1 (potent vasoconstrictor) and other cytotoxic factors, which leads to repeated myocardial ischaemia and myocardial fibrosis. Autonomic nervous system dysfunction also contributes to intermittent microvascular spasm. In the immune-mediated inflammatory pathway, adhesion molecules facilitate the migration of lymphocytes into the extracellular matrix, where CD4 on T cells and auto-antibodies (found in SSc) activate fibroblasts to become myofibroblasts which secrete collagen, causing myocardial fibrosis. Interleukin IL-1α, released after tissue damage or cell death, induces interleukin IL-6 and platelet-derived growth factor A (PDGFA), both of which activate myofibroblasts to secrete collagen. Increased levels of NLRP3 inflammasome (NOD-, LRR- and pyrin domain-containing protein 3), found in systemic sclerosis (SSc) fibroblasts, activate interleukin IL-1β, which then synergizes with transforming growth factor beta 1 (TGF-1β) to stimulate endothelial to mesenchymal transition (EndMT) and promote fibrogenesis. Created in BioRender. Soo, C. (2025) https://BioRender.com/8z94fux

The pathophysiology of SSc-PHI is complex and remains incompletely understood. It is generally thought that SSc-PHI is driven by vascular and immune mediated pathways, exacerbated by the NLRP3 inflammasome and mediated by cytokines, which jointly orchestrate myocardial fibrogenesis (Fig. 1). However, most of the proposed mechanisms discussed below remain hypothetical and are extrapolated from broader studies of SSc fibrosis or systemic vascular dysfunction [17].

### Vascular pathway

The ‘vascular hypothesis’ of fibrogenesis proposes intermittent vascular spasm or ‘intramyocardial Raynaud’s phenomenon’ as a mechanism leading to myocardial fibrosis. In an autopsy study of 52 SSc patients, 23 (44%) had myocardial lesions (contraction band necrosis to replacement fibrosis), despite morphologically normal intramyocardial and epicardial coronary arteries [18]. More severe histological lesions correlated with clinical ventricular arrhythmias, congestive heart failure, angina and sudden death [18]. In another autopsy study, 31/58 (53%) of SSc patients had patchy myocardial fibrosis without significant atherosclerosis in epicardial coronary arteries [19]. The notion that repeated myocardial ischaemia due to microvascular impairment drives myocardial fibrosis in SSc was further supported by clinical imaging studies [20–24].

Beyond vasospasm, indirect evidence from general SSc cohorts supports the profibrotic role of different vascular mediators and cytotoxic factors [25, 26]. These include endothelin-1(a potent vasoconstrictor) and superoxide anions which inactivate the vasodilator nitric oxide and oxidize circulating low-density lipoproteins (LDLs), increasing vascular susceptibility to free radical damage [25–27]. Cytotoxic factors directly detrimental to endothelial cells have been isolated from SSc sera including anti-endothelial cell antibodies, endothelial cell growth-inhibitory factor and complement membrane attack complex (C5b-C9) [28–30]. They likely synergize to cause intermittent vascular spasm, ischaemic myocardial necrosis, reperfusion injury and eventually fibrogenesis [31].

### Immune-mediated inflammatory pathway

Recent studies highlight the prominent role of immune-mediated inflammatory processes in SSc^32^-PHI. Increased levels of adhesion molecules facilitate the migration of lymphocytes [T, B cells and natural killers (NK) cells] through the endothelium into the extracellular matrix [33]. Both the vascular cell adhesion molecule-1 (VCAM-1) and endothelial leucocyte adhesion molecule-1 (E-selectin) circulating levels correlate positively with the severity of skin score and renal function in SSc [32]. These mononuclear immune cells adhere so well to the endothelium that their levels are reduced in the peripheral blood in SSc *vs* healthy controls [34]. In the extracellular matrix, T cells display different markers including CD4, activating fibroblasts to become myofibroblasts which secrete collagen [35, 36]. Approximately 95% of SSc patients have at least one circulating autoantibody [37], which further stimulate fibroblasts to secrete collagen by either upregulating their intercellular adhesion molecule-1 (ICAM-1), promoting secretion of proinflammatory cytokines, activating the platelet-derived growth factor (PDGF) receptors on fibroblasts or binding directly to them [38–40].

### Cytokines and inflammasome

Inflammatory cytokines play a cardinal role in the myocardial inflammatory cascade and the development of cardiac dysfunction. The interleukin-1 (IL-1) family of cytokines has 11 individual members which modulate acute phase inflammation by binding to receptors and co-receptors with either pro- or anti-inflammatory function [41]. IL-1α is released following ischaemic endothelial cell death or tissue damage [42]. It induces IL-6 and platelet-derived growth factor A (PDGFA), triggering inflammation, fibroblast proliferation and differentiation into myofibroblasts with increased collagen production [43]. The inactive form of IL-1β is cleaved and activated by inflammasomes, the intracellular multimeric protein complexes involved in the caspase-1 inflammatory response, which modulate apoptosis [44]. Activated IL-1β synergizes with transforming growth factor beta 1 (TGF-1β) to stimulate endothelial to mesenchymal transition (EndMT) and promote fibrogenesis, as seen in the cardiac and pulmonary tissues of mouse models [45, 46]. SSc fibroblasts exhibit increased levels of NLRP3 inflammasome (NOD-, LRR- and pyrin domain-containing protein 3) and micro-RNA (miR)-155 essential for collagen synthesis [47, 48]. Depletion of miR-155 reduced cardiac fibrosis in mice subjected to pressure overload [49].

In experimental studies, IL-1 directly depressed left ventricular (LV) function in mice injected with serum from patients with heart failure or septic shock [50–53]. Additionally, IL-17, secreted by T helper cells (TH17), contributed to post-myocarditis cardiac fibrosis and dilated cardiomyopathy, in synergy with IL-1 [54, 55].

### Cardiac autonomic nervous system dysfunction

Cardiac autonomic nervous system dysfunction occurs early in SSc [56, 57]. In practice, it can be detected by reduced heart rate variability (HRV) on 24-h Holter monitor and abnormal responses to Ewing’s autonomic tests [56]. In a prospective study, 13/26 (50%) SSc without cardiovascular (CV) symptoms had cardiac autonomic nervous system dysfunction which correlated with an active pattern on nailfold video-capillaroscopy [58]. Another study showed independent association between reduced HRV and subclinical cardiac impairment (reduced global longitudinal strain, GLS and global circumferential strain, GCS on echocardiography) [59]. These observations suggest that autonomic nervous system dysfunction may contribute to SSc-PHI by promoting vasospasm and subsequent microvascular injury.

## Manifestations of SSc-PHI

**Figure 2:**
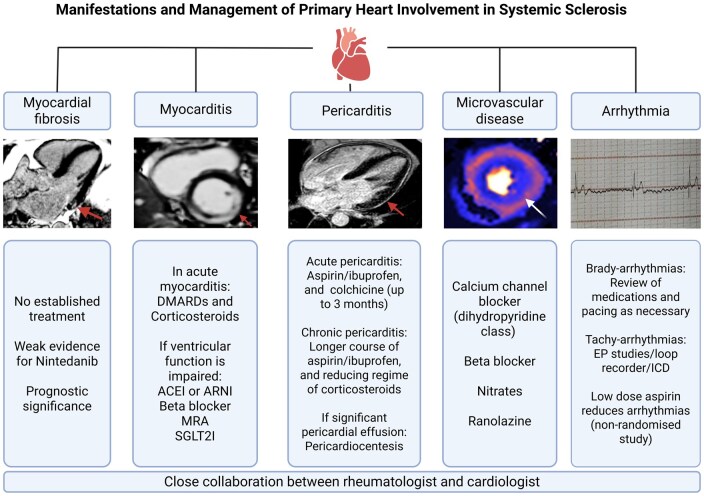
Management of primary heart involvement in systemic sclerosis. Clinical manifestations of primary heart involvement in systemic sclerosis (SSc) include: myocardial fibrosis [red arrow pointing to mid-wall fibrosis (white) in the basal infero-lateral segment on cardiac magnetic resonance imaging (CMR) with gadolinium]; myocarditis (red arrow on CMR image pointing to mid-wall late gadolinium hyperenhancement in the basal infero-septum, inferior and lateral segments); Pericarditis [red arrow pointing to pericardial hyperenhancement (white) on CMR]; microvascular disease (quantitative perfusion on CMR showing circumferentially reduced subendocardial myocardial blood flow with adenosine stress); arrhythmia (electrocardiogram rhythm strip showing complete heart block on background of atrial fibrillation). ACEI, angiotensin-converting enzyme inhibitor; ARNI, angiotensin receptor-neprilysin inhibitor; DMARDs, disease-modifying anti-rheumatic drugs; EP studies, electrophysiological studies; ICD, implantable cardioverter-defibrillator; MRA, mineralocorticoid receptor antagonist; SGLT2i, sodium-glucose cotransporter-2 inhibitor. Created in BioRender. Soo, C. (2025) https://BioRender.com/6z7snix

The prevalence of primary cardiac involvement in SSc varies between 15% and 35%, with subclinical SSc-PHI thought to be much more common [5]. Predictors for cardiac involvement include demographic factors (male gender, Black/Eastern European ethnicity, onset ≥65 years old), diffuse subtype of SSc, certain physical signs (rapidly progressing skin disease, digital ulcers, tendon friction rubs), positive serum auto-antibodies (especially anti-U3-RNP and anti-SCL70), pulmonary involvement, peripheral myositis and late pattern on nailfold video-capillaroscopy [8, 21, 60–63]. The most common manifestations of SSc-PHI are myocarditis, pericarditis, conduction abnormalities, micro- and macrovascular coronary artery disease, and the final pathway of LV and RV contractile dysfunction (Table 1, Fig. 2).

**Table 1:** Manifestations, symptoms, diagnostic approach and management of SSc-PHI.

Manifestation	Symptoms	Diagnosis	Management
Myocarditis	Often subclinical Chest pain dyspnoea Fatigue Palpitations	Serum high sensitive troponin Serum NT-proBNP ECG Echocardiogram CMR	Immunosuppressants Corticosteroids (cave scleroderma renal crisis) Management of complications
Pericardial disease	Pleuritic chest pain, dyspnoea, peripheral swelling	ECG Chest X-ray Serial echocardiograms CMR Cardiac CT	**Acute pericarditis:** Anti-inflammatory medication Colchicine for up to 3 months **Recurrent pericarditis:** Corticosteroids Longer-term immunosuppressants Intravenous immunoglobulin Anakinra Azathioprine **Large effusion with haemodynamic compromise:** Cardiology review Pericardiocentesis Pericardial window as last resort
Heart failure	Exertional dyspnoea Paroxysmal nocturnal dyspnoea, Peripheral oedema	Serum NT-proBNP Serum high-sensitive troponin ECG Chest X-ray Echocardiogram Consider CMR	**Acute presentation:** Cardiology admission Intravenous diuretics **Chronic presentation:** ARNI/ARB/ACEI BBs with caution MRA, SGLT-2 inhibitors **Diastolic HF:** SGLT-2 inhibitor MRA
Conduction system disease (brady-arrhythmias)	Syncope Pre-syncope Light-headedness	Serum electrolytes Thyroid function tests ECG Ambulatory ECG Loop recorder	Cardiology review Medication review (consider stopping BB or CCB) Pacemaker for high degree AV block
Tachy-arrhythmias	Palpitations Syncope	Serum electrolytes Thyroid function tests ECG Ambulatory ECG Loop recorder	Cardiology review Electro-physiological studies BB other anti-arrhythmic drugs Ablation Defibrillator
Microvascular coronary artery disease	Angina-like chest pain	Serum high sensitive troponin ECG Echocardiogram CMR or PET with (quantitative) stress perfusion Cardiac CT angiogram to rule out epicardial CAD	Cardiology review Calcium channel blockers Beta blockers Nitrates Ranolazine Consider combining BB with CCB to reduce Raynaud’s phenomenon

ACEI, angiotensin-converting enzyme inhibitor; ARNI, angiotensin receptor-neprilysin inhibitor; ARB, angiotensin receptor blocker; AV, atrioventricular; BBs, beta-blockers; CAD, coronary artery disease; CCB, calcium channel blocker; CMR, cardiac magnetic resonance; CT, computed tomography; ECG, electrocardiogram; MRA, mineralocorticoid receptor antagonist; NT-proBNP, N-terminal pro-B-type natriuretic peptide; PET, positron emission tomography; SGLT-2, sodium-glucose cotransporter-2 inhibitor.

### Myocarditis

Myocardial inflammation as a manifestation of SSc-PHI is common and often subclinical. Symptoms of myocarditis are highly variable, ranging from chest discomfort and lethargy in mild instances to symptoms of overt heart failure (dyspnoea, leg swelling), syncope or even death (from arrhythmias or ventricular failure) in fulminant myocarditis [64]. Acute SSc myocarditis is typically more severe than other forms of myocarditis. A retrospective study involving three Italian centres analysed endomyocardial biopsy-proven non-viral myocarditis, satisfying the Dallas criteria, in three cohorts of patients: SSc, other autoimmune diseases and isolated non-viral myocarditis [65]. SSc myocarditis was significantly more likely to present with dyspnoea, clinical cardiac failure, had higher frequency of ventricular ectopics on 24-h electrocardiogram (ECG) and more severe myocardial fibrosis on histological analysis [65]. The latter correlated positively with the modified Rodnan skin score and mortality between 12 and 60 months post-myocarditis [65]. At the cellular level, the release of IL-1α from injured myocytes, precipitated by tissue damage, sets in motion a downstream inflammatory cascade involving other mediators including IL-6, TGF-β and inflammasome, leading to myocardial inflammation, potentially apoptosis, fibrosis and eventually cardiac dysfunction [31, 66].

### Pericardial disease

Pericardial involvement is common in SSc and can be a manifestation of primary heart involvement, often concomitant with myocarditis as a myopericardial inflammatory syndrome, or secondary to other SSc organ manifestations, including PAH and renal disease [67]. Acute pericarditis usually presents with positional pleuritic chest pain, pyrexia or dyspnoea, with or without pericardial effusion [68]. Chronic pericarditis may lead to pericardial constriction, where the diastolic expansion of the cardiac chambers becomes restricted by fibrosed pericardium, causing features of right heart failure [69]. Features of focal, diffuse fibrous or fibrinous pericarditis were evident in 33–78% of SSc patients in two autopsy studies [18, 67]. A prospective echocardiography study detected pericardial effusion in 41% (22/54) SSc patients without correlation to PAH [70]. Most pericardial effusions (82%) were small to moderate (<200 mL) and asymptomatic [70]. Another prospective study showed asymptomatic pericardial effusion in 15% (15/100) of SSc patients without PAH [71]. An SSc autopsy series identified higher prevalence of pericardial disease (fibrinous adhesions or effusion >50 mL) in 19/34 (56%) without correlation to PAH [72]. In a 10-year retrospective study, 6.9% (32/462) of SSc patients were admitted with symptomatic pericardial effusion, of whom 47% and 74% had concurrent RV failure and PAH, respectively [73]. Severe pericardial effusion (very large >1 litre or rapidly accumulating) can cause cardiac tamponade, a medical emergency with high mortality, when increased intrapericardial pressure exceeds the elastic limit of the pericardium, compressing the heart chambers [74]. A review of 25 SSc patients, without PAH, diagnosed with cardiac tamponade at presentation recorded significant mortality (12.5%) despite treatment [75]. In a prospective study of 335 SSc patients with concurrent PAH, persistent pericardial effusion was associated with reduced survival compared with those who never had pericardial effusion [76].

### Conduction system disease

Microvascular ischaemic injury and subsequent fibrosis in the cardiac conduction system provides substrate for a range of cardiac arrhythmias [77]. The annual incidence of sudden cardiac death (SCD) in SSc of 1.0–3.3% is >10 times higher than in the general population (0.05–0.1%) [78, 79]. The most common arrhythmias in SSc are ventricular ectopic beats (VEBs, 70%), non-sustained ventricular tachycardia (NSVT, 18%) and atrial fibrillation (AF, 8%) [78]. Increased replacement myocardial fibrosis (detected as mid-wall late gadolinium enhancement, LGE), diffuse myocardial fibrosis (measured as increased extracellular volume, ECV) and increased T1 value on CMR correlated with abnormal arrhythmias on both Holter and implantable loop recorders [65, 80–83]. In a prospective study, a threshold of >1190 VEBs in 24 h predicted sudden cardiac death or need for implantable cardiac defibrillator (ICD) in SSc without PAH [84]. Right bundle branch block (RBBB) and severe ventricular arrhythmias were independently associated with increased risk of SCD [77, 78]. The risk of SCD was even higher (*n* = 12/25) in SSc patients who had concomitant skeletal myopathy.

### Ventricular impairment

Primary LV diastolic dysfunction in SSc had a prevalence of 18–23% in echocardiography studies, which either excluded patients with PAH or adjusted for PAH and ILD, and was independently associated with mortality [85, 86]. LV diastolic dysfunction is thought to be the result of progressive myocardial fibrosis which causes LV stiffness with impaired relaxation, ultimately leading to clinical heart failure as restrictive cardiomyopathy [87]. The risk factors independently associated with LV diastolic dysfunction were SSc disease duration, age, systemic hypertension and co-existing coronary artery disease, hence the importance of managing modifiable traditional CV risks [85].

Overt LV systolic dysfunction is less common (5.3% in EUSTAR database) in SSc, but is independently associated with male gender, age, digital ulcerations, myositis and non-use of calcium channel blockers [63, 88]. In a retrospective study of SSc patients without CV symptoms or PAH, CMR identified sub-clinical reduction in LV GLS, despite normal bi-ventricular ejection fractions (EFs) [89]. Reduced LV GLS was independently predictive of mortality, affirming its role as a more sensitive marker of subtle cardiac dysfunction in SSc [89]. When SSc patients with PAH and ILD were included in a broader retrospective cohort, both increased native T1 value and reduced RVEF independently predicted mortality [90]. When adjusted for PAH, three phenotypic clusters on CMR that were predictive of mortality were RV failure, bi-ventricular failure and dilated cardiac chambers despite normal contractile function [90].

RV systolic dysfunction can be caused by primary RV disease or occur secondary to PAH. The concept of primary RV involvement in SSc is supported by several prospective studies which demonstrated subclinical RV systolic and diastolic impairment in cohorts without PAH [13, 91, 92]. An *ex vivo* biopsy study showed abnormal RV sarcomere function in SSc without PAH, suggesting that primary RV dysfunction in SSc can occur irrespective of PAH [93]. While impairment in RV systolic dysfunction in the absence of PAH is commonly subclinical, clinically relevant and more severe RV disease is seen in SSc with PAH [13].

PAH from ILD may lead to reduced RV systolic function (cor pulmonale) associated with 3-year survival of 52% from diagnosis of PAH in a meta-analysis [94, 95]. In addition, both cyclophosphamide (at myeloablative dose) and anthracycline (at cumulative dose of >300 mg/m^2^), used in haematological stem cell transplantation for SSc can also cause reduced LVEF [96–98].

### Microvascular and epicardial coronary artery disease

In SSc, the coronary micro-circulation is particularly vulnerable to insults from vascular mediators, cytotoxic factors and vasospasm [99]. Inadequate blood flow through the micro-circulation to meet cardiac metabolic demands causes CMD or microvascular angina with symptoms almost indistinguishable from epicardial coronary artery disease [100].

Transient myocardial ischaemia due to peripheral cold exposure (cardiac Raynaud’s phenomenon) was identified on thallium-201 single photon emission computed tomography (SPECT) in SSc patients without clinical PHI [101, 102]. In a prospective study using cold provocation test and contrast echocardiography, 29.4% (*n* = 15) of SSc patients without clinical PHI had severe cardiac Raynaud’s phenomenon, which predicted deterioration in LVEF over 7 years of follow-up [103].

Unlike microvascular disease, most previous studies have shown similar prevalence of epicardial coronary artery disease in SSc *vs* the general population [104]. However, a recent systematic review found that the risk of myocardial infarction in SSc was nearly double compared with a control group, suggesting combined cumulative risks over time from vascular injury, chronic inflammation and autoimmunity [16].

### Accelerated atherosclerosis

Whilst microvasculopathy is the hallmark of SSc-PHI, it is increasingly being recognized that the prevalence of macrovascular disease is also increased in SSc, involving peripheral arteries, cerebrovascular vessels, carotid arteries and epicardial coronary arteries, implicating accelerated atherosclerosis [15]. This, along with SSc-PHI, adds to the overall CV burden in SSc [15, 16]. Although there is no prospectively validated risk score for CV disease specific for SSc to account for this excess risk, when the systemic lupus erythematosus (SLE) option in QRISK3 was applied as a surrogate for SSc, it estimated a higher proportion of patients at high risk of cardiovascular disease (CVD) compared with Framingham Risk Score [105]. Modifiable risk factors accounted for 79.6% of global CVD burden, reinforcing the need for aggressive control of traditional CV risk factors, alongside SSc-specific management [106].

## Diagnosing cardiac involvement in SSc

Many patients with SSc-PHI are asymptomatic. The World Scleroderma Foundation/Heart Failure Association consensus statement for SSc-PHI therefore advocates regular screening for PHI in all SSc patients, emphasizing prompt cardiology input, where indicated [7, 31]. The standard diagnostic work-up at new SSc diagnosis thence annually, including in asymptomatic patients, should include history, examination, 12-lead ECG, serial serum biomarkers of cardiac involvement (hs-troponin, NT-proBNP) and a transthoracic echocardiogram [7].

SSc patients may present with a range of CV symptoms including angina-like chest pain (precipitated by physical exertion or exposure to cold weather), palpitations, syncope or pre-syncope, exertional dyspnoea, orthopnoea, paroxysmal nocturnal dyspnoea (PND), postural hypotension, pleuritic chest pain, peripheral swelling or abdominal distension [107]. The severity of SSc-PHI can be graded using a scoring system (Table 2). CV symptoms should prompt repeat ECG or Holter monitor (24 h or longer) and blood biomarkers [107]. Transthoracic echocardiography is generally the first-line imaging tool. However, the quality of echocardiography, particularly for RV imaging, may be variable, and is more dependent on body habitus than with cross-sectional imaging techniques [108].

**Table 2:** Modified Medsger severity score for cardiac involvement in SSc.

Score	Criteria
0 (normal)	Normal ECG; LVEF ≥50%
1 (mild)	Conduction defects on ECG; LVEF 45–49%
2 (moderate)	Arrhythmia on ECG; LVEF 40–44%
3 (severe)	Arrhythmia requiring treatment; LVEF 30–40%
4 (end-stage)	Congestive heart failure; LVEF <30%

Adapted from Medsger et al. [61].

ECG, electrocardiogram; LVEF, left-ventricular ejection fraction.

CMR is now recommended when SSc-PHI is suspected following initial assessment [7]. CMR allows accurate assessment of structure and function of all cardiac chambers, including the RV, pericardium and detailed myocardial tissue characterization including oedema, diffuse and focal fibrosis and ischemia [108]. CMR is particularly useful in suspected immune-mediated myocarditis, to accurately assess bi-ventricular function and quantify myocardial fibrosis. The decision to refer for CMR may be supported by ‘red flags’ of elevated blood CRP and troponin levels, as well as the presence of ILD and arrhythmias on 24 h Holter according to a recent retrospective study in which these clinical findings were independently associated with abnormal CMR in patients with SSc-PHI [81].

Where available, PET CT can also be used to detect myocardial and vascular inflammation and ischaemia. 18F FDG PET has been used to track inflammation in SSc myocarditis [65] and the new PET tracer 68Ga Fibroblast Activation Protein Inhibitor (FAPI) may track fibrogenesis in the heart [109].

It is, however, important to note that findings on CMR and PET/CT are generally non-specific, and no imaging biomarkers exist to definitively identify SSc-related CV disease. In particular, in the absence of specific diagnostic criteria for immune-mediated myocarditis, in practice diagnosis is based on guidance developed for viral myocarditis, which may lack sensitivity and specificity in SSc [110]. Furthermore, widely accepted standards for imaging acquisition and interpretation in SSc and other immune-mediated inflammatory diseases (IMIDs) are lacking.

Endomyocardial biopsy may be used to evaluate cases of myocarditis and cardiomyopathy where the aetiologies remain unclear despite non-invasive investigations [111]. When occasionally used, biopsy provides insights into the cellular mechanisms of SSc-PHI [65].

## Management of cardiac involvement in SSc

Treatments are guided by the nature of cardiac involvement and best overseen by a multi-disciplinary team comprising rheumatologists and cardiologists experienced in the management of SSc and its CV manifestations (Fig. 2). However, there is a limited evidence base for the management of SSc-PHI with data derived from small case series or cohort studies (summarized by Batani *et al.*). [112] Controlling SSc disease activity in general is paramount in patients with cardiac manifestations, combined with supportive therapies and interventions in line with other, non-immune mediated cardiovascular diseases [107]. Based on the current literature, among the available immunosuppressants, mycophenolate (MMF) will often be the first choice to treat suspected SSc-PHI, with biologics such as tocilizumab and rituximab potential alternatives [112]. In addition, calcium channel blockers or vasodilators are commonly added [112].

### Myocarditis

There is a very limited evidence base for the use of immunosuppressants in the management of SSc myocarditis. In one small study (*n* = 18) symptomatic SSc-myocarditis was treated with immunosuppressants (cyclophosphamide/rituximab, MMF, tocilizumab or methotrexate), corticosteroids alone or autologous stem cell transplantation [113]. In response to treatment, myocardial oedema on CMR reduced and cardiac function improved, while native T1 and ECV remained unchanged. Another study of 35 SSc-PHI patients from two centres showed reduction in myocardial T1 and T2 on CMR, indicative of reduced myocardial inflammation in response to 12 month treatment with a range of immunosuppressants (including MMF, cyclophosphamide and biologics) [114]. These changes were paralleled by a reduction in cardiac biomarkers (NT-proBNP, hsTnI) and CRP. And in a small study of asymptomatic SSc patients (*n* = 9) with subclinical myocarditis, CMR showed resolution of myocardial oedema and ECV at 6 months using prednisolone 20 mg and azathioprine 100 mg once daily [115]. Despite these initial studies suggesting improvement in myocardial oedema/inflammation in response to immunosuppressive therapy, larger randomized controlled trials are required to clarify optimal management of SSc myocarditis.

Both IL-1 and IL-6 play pivotal roles in immune-mediated myocarditis and could represent potential therapeutic targets [51]. A case report showed resolution of myocardial oedema with IL-6 inhibitor tocilizumab in SSc myocarditis [116]. Other agents including rilanocept, anakinra and rituximab have theoretical benefits in treating immune-mediated myocarditis but currently lack specific evidence [117–119].

Corticosteroids (>15 mg/day) can precipitate scleroderma renal crisis especially in diffuse SSc and those with positive anti-RNA polymerase III antibodies [120]. Cyclophosphamide at high doses (in haematopoietic stem cell transplantation) risks cardiotoxicity [96–98]. Therefore, their use necessitates extreme caution and close monitoring of renal and cardiac functions, respectively.

### Pericardial disease

Asymptomatic mild (<10 mm) to moderate pericardial effusion (10–20 mm) without imaging markers of haemodynamic compromise may be monitored with transthoracic echocardiogram regularly [118]. Pericardiocentesis is recommended for larger or symptomatic pericardial effusion or for diagnostic purposes to assess for concurrent bacterial or neoplastic aetiology [118]. Surgical pericardial windows can be considered as a last resort for recurrent large pericardial effusion with haemodynamic compromise but it carries high risks of mortality in the presence of PAH [121, 122].

Acute pericarditis can be treated with oral aspirin or ibuprofen for 1–2 weeks and colchicine for up to 3 months [118]. However, evidence for their efficacy in SSc is lacking. Chronic or recurrent pericarditis, without obvious aetiology, is generally considered immune-mediated [118, 123]. It is treated with a longer course of aspirin, ibuprofen or indomethacin, and colchicine with gradual withdrawal [118]. Corticosteroids may be used at low doses (0.2–0.5 mg/kg/day) in the absence of bacterial infections, especially tuberculosis, and with very slow decrements of 1–2.5 mg every 2–6 weeks [118]. Exercise restrictions should be considered until symptoms and CRP have resolved [118]. Intravenous immunoglobulin (IVIG), rilonacept or anakinra (interleukin-1 receptor antagonists) or azathioprine may be used in recurrent pericarditis not responsive to colchicine [118].

Pericarditis of any cause may lead to constrictive pericarditis which often requires pericardiectomy [118]. However, there were reports of transient constriction which had resolved with empiric anti-inflammatory drugs [124, 125].

### Conduction defects and tachyarrhythmias

There is no specific guideline for management of SSc arrhythmias and general cardiology guidelines apply which are well-evidenced. In bradyarrhythmias, medications should be withheld which slow the atrio-ventricular (AV) node conduction including beta-blockers, non-dihydropyridine calcium channel blockers or other anti-arrhythmic agents. Persistent higher degree AV node block, symptomatic bradycardia, sinus node disease or chronotropic incompetence may need treatment with permanent pacemaker implantation [126]. Tachy-arrhythmias may require interrogation by electro-physiological studies and are managed according to their mechanisms and risk-stratification [127, 128]. Treatments include beta-blockers – with caution due to their potential vasospastic effects – other anti-arrhythmic drugs, ablation therapy or implantation of a defibrillator [127, 128]. The CHA2DS2-VASc score is used to assess stroke risk in non-valvular atrial fibrillation to guide decision on anticoagulation [129]. In a non-randomized observational study, low-dose aspirin (≤325 mg daily) was associated with lower incidence of heart block or eventual pacemaker implantation [130]. However, its use needs to be balanced against bleeding risks, especially in SSc with gastrointestinal involvement.

### Heart failure (HF)

As in non-SSc patients, the priorities in treating heart failure in patients with SSc are the management of symptoms, alleviating volume overload, mitigating precipitating causes and improving patient outcomes. Acute decompensated heart failure requires hospitalization and monitored diuresis with intravenous diuretics [131]. In chronic heart failure with reduced LVEF, European Society of Cardiology guidelines advocate four pillars of medical treatments (Class I indication): angiotensin-converting enzyme inhibitor (ACEI) or angiotensin receptor neprilysin inhibitor (ARNI) with angiotensin II receptor blocker (sacubitril/valsartan), beta-blocker (B blocker), mineralocorticoid receptor antagonists (MRA), sodium-glucose cotransporter 2 (SGLT-2) inhibitor, which independently improve prognosis [131]. Cardiac re-synchronization therapy (CRT) is recommended for symptomatic HF patients with LVEF ≤ 35%, QRS duration ≥150 ms and left bundle branch block (LBBB) morphology on ECG, despite optimal medial therapy, to reduce morbidity or mortality [131]. Implantation of a cardioverter-defibrillator in HF patients, fulfilling certain criteria, is recommended for primary or secondary prevention of SCD [131]. Challenging decisions on these treatments are evaluated by local heart multi-disciplinary teams.

SGLT-2 inhibitors (Class I recommendation) reduce hospitalizations and improve prognosis in heart failure with preserved EF (diastolic dysfunction) [132]. The non-steroidal MRA inhibitor, finerenone, was recently found to improve prognosis and reduce hospitalizations in heart failure with either mildly reduced or preserved EF (LVEF ≥50%) [133]. There are no specific prognostic data of these agents in SSc-specific cohorts.

There are no established treatments for myocardial fibrosis, which is thought to be the precursor to heart failure. Nintedanib is an anti-fibrotic agent which competitively inhibits the tyrosine kinase domains on vascular endothelial growth factor (VEGF), PDGF and fibroblast growth factor (FGF) [134]. These growth factors are indispensable to fibrogenesis in SSc and ILD [135]. In a small study of 20 patients with SSc-ILD, nintedanib significantly reduced myocardial ECV on CMR, indicative of reduced diffuse myocardial fibrosis, suggesting a possible anti-fibrotic effect in the heart [136].

### Microvascular angina

Microvascular angina can be treated with calcium channel blockers, beta blockers, nitrates or ranolazine [137]. Oral nifedipine 60 mg once daily improved myocardial perfusion reserve on CMR over a 14-day period [138]. In a multicentre observational study, vasodilator therapy (nifedipine up to 60 mg, or captopril up to 100 mg, once daily) correlated with lower incidence of ventricular arrhythmias (*P*= 0.03) over 4 years of follow-up [130]. Potential adverse effects of beta-blockers on Raynaud’s phenomenon may be ameliorated with concurrent administration of beta blockers with dihydropyridine calcium channel blockers [139]. The latter primarily act on vascular smooth muscles rather than cardiac receptors to minimize risks of bradycardia [140].

## Unmet needs and potential solutions in SSc-PHI

Despite much progress in the management of SSc in general, SSc-PHI remains under-researched and CV mortality in SSc remains high. There is an unmet need to better understand the vascular, inflammatory and immune-mediated mechanisms that drive PHI in SSc. How existing and emerging biologic therapeutics impact CV involvement in SSc is largely unknown. Additionally, the advancement of multi-omic platforms which reveal intricate correlations between genetic predispositions, immune dysregulation and environmental triggers in the pathogenesis of SSc, offers opportunities for new therapeutic targets and more personalized treatments. Early detection of subclinical CV disease in SSc is perhaps the most important unmet need to improve risk stratification and CV outcome. Developing reliable diagnostic pathways, including the appropriate use of current and new humoral and imaging biomarkers for SSc-PHI is timely. This is also a prerequisite for the development and testing of specific therapies targeting SSc-PHI, which currently are lacking. Most importantly, multicentre studies and registries with larger number of SSc patients are warranted to validate the findings of studies with smaller number of patients to better understand the prognostic implications of subclinical disease detected on imaging.

## Conclusions

SSc-PHI is common and is associated with significantly adverse outcomes. The underlying mechanisms of SSc-PHI are complex and incompletely understood. As an evidence base for the prevention and management of SSc is largely lacking, patient care is inconsistent and undoubtedly suboptimal in many cases. Several programmes are in development to address this limitation, including the CARDIO-IMID network in the UK, that will provide critical data for the best use of screening and advanced diagnostic tools in the coming years. The increasingly close collaboration of cardiologists and rheumatologists in many healthcare systems will aid in providing inter-disciplinary care for patients with SSc.

## Data Availability

No new scientific data were generated or analysed in support of this review. All information discussed is drawn from previously published studies cited in the article. The interpretations and views presented are those of the authors.

## References

[1] van den Hoogen F , Khanna D, Fransen J et al 2013 classification criteria for systemic sclerosis: an American college of rheumatology/European league against rheumatism collaborative initiative. Ann Rheum Dis 2013;72:1747–55. doi: 10.1136/annrheumdis-2013-204424 24092682

[2] Airo P , Regola F, Lazzaroni MG et al Incidence and prevalence of systemic sclerosis in Valcamonica, Italy, during an 18-year period. J Scleroderma Relat Disord 2020;5:51–6. doi: 10.1177/2397198318819908 35382405 PMC8922587

[3] Fernández-Ávila DG , Bernal-Macías S, Gutiérrez JM, Rincón DN, Rosselli D. Prevalence of systemic sclerosis in Colombia: data from the National Health Registry 2012-2016. J Scleroderma Relat Disord 2020;5:137–42. doi: 10.1177/2397198319873526 35382022 PMC8922607

[4] Bairkdar M , Rossides M, Westerlind H et al Incidence and prevalence of systemic sclerosis globally: a comprehensive systematic review and meta-analysis. Rheumatology (Oxford) 2021;60:3121–33. doi: 10.1093/rheumatology/keab190 33630060 PMC8516513

[5] Pokeerbux MR , Giovannelli J, Dauchet L et al Survival and prognosis factors in systemic sclerosis: data of a French multicenter cohort, systematic review, and meta-analysis of the literature. Arthritis Res Ther 2019;21:86. doi: 10.1186/s13075-019-1867-1 30944015 PMC6446383

[6] Elhai M , Meune C, Boubaya M, EUSTAR group et al Mapping and predicting mortality from systemic sclerosis. Ann Rheum Dis 2017;76:1897–905. doi: 10.1136/annrheumdis-2017-211448 28835464

[7] Bruni C , Buch MH, Djokovic A et al Consensus on the assessment of systemic sclerosis-associated primary heart involvement: world Scleroderma Foundation/Heart Failure Association guidance on screening, diagnosis, and follow-up assessment. J Scleroderma Relat Disord 2023;8:169–82. doi: 10.1177/23971983231163413 37744047 PMC10515996

[8] Bruni C , Buch MH, Furst DE et al Primary systemic sclerosis heart involvement: a systematic literature review and preliminary data-driven, consensus-based WSF/HFA definition. J Scleroderma Relat Disord 2022;7:24–32. doi: 10.1177/23971983211053246 35386946 PMC8922675

[9] Kahan A , Coghlan G, McLaughlin V. Cardiac complications of systemic sclerosis. Rheumatology (Oxford). 2009;48(Suppl 3):iii45-48. doi: 10.1093/rheumatology/kep110 19487224

[10] Bruni C , Ross L. Cardiac involvement in systemic sclerosis: getting to the heart of the matter. Best Pract Res Clin Rheumatol 2021;35:101668. doi: 10.1016/j.berh.2021.101668 33736950

[11] Bissell LA , Md Yusof MY, Buch MH. Primary myocardial disease in scleroderma-a comprehensive review of the literature to inform the UK Systemic Sclerosis Study Group cardiac working group. Rheumatology (Oxford) 2017;56:882–95. doi: 10.1093/rheumatology/kew364 27940590

[12] Bairkdar M , Dong Z, Andell P, Hesselstrand R, Holmqvist M. POS1286 arrhythmia in patients with systemic sclerosis: a Swedish register-based study. Ann Rheum Dis 2023;82:989–90. doi: 10.1136/annrheumdis-2023-eular.2861 PMC11337701 39164050

[13] Tedford RJ , Mudd JO, Girgis RE et al Right ventricular dysfunction in systemic sclerosis-associated pulmonary arterial hypertension. Circ Heart Fail 2013;6:953–63. doi: 10.1161/CIRCHEARTFAILURE.112.000008 23797369 PMC3815697

[14] Martin M , Fornecker LM, Marcellin L et al Acute and fatal cardiotoxicity following high-dose cyclophosphamide in a patient undergoing autologous stem cell transplantation for systemic sclerosis despite satisfactory cardiopulmonary screening. Bone Marrow Transplant 2017;52:1674–7. doi: 10.1038/bmt.2017.188 28869615

[15] Cannarile F , Valentini V, Mirabelli G et al Cardiovascular disease in systemic sclerosis. Ann Transl Med 2015;3:8. doi: 10.3978/j.issn.2305-5839.2014.12.12 25705640 PMC4293487

[16] Chen IW , Wang WT, Lai YC et al Association between systemic sclerosis and risk of cerebrovascular and cardiovascular disease: a meta-analysis. Sci Rep 2024;14:6445. doi: 10.1038/s41598-024-57275-9 38499699 PMC10948904

[17] Allanore Y , Meune C. Primary myocardial involvement in systemic sclerosis: evidence for a microvascular origin. Clin Exp Rheumatol. 2010;28:S48-53. 21050545

[18] Bulkley BH , Ridolfi RL, Salyer WR, Hutchins GM. Myocardial lesions of progressive systemic sclerosis. A cause of cardiac dysfunction. Circulation 1976;53:483–90. doi: 10.1161/01.cir.53.3.483 1248080

[19] D’Angelo WA , Fries JF, Masi AT, Shulman LE. Pathologic observations in systemic sclerosis (scleroderma). A study of fifty-eight autopsy cases and fifty-eight matched controls. Am J Med 1969;46:428–40. doi: 10.1016/0002-9343(69)90044-8 5780367

[20] Faccini A , Agricola E, Oppizzi M et al Coronary microvascular dysfunction in asymptomatic patients affected by systemic sclerosis - limited vs. diffuse form. Circ J 2015;79:825–9. doi: 10.1253/circj.CJ-14-1114 25740209

[21] Dumitru RB , Bissell L-A, Erhayiem B et al Predictors of subclinical systemic sclerosis primary heart involvement characterised by microvasculopathy and myocardial fibrosis. Rheumatology (Oxford) 2021;60:2934–45. doi: 10.1093/rheumatology/keaa742 34080001 PMC8213428

[22] Sulli A , Ghio M, Bezante GP et al Blunted coronary flow reserve in systemic sclerosis. Rheumatology (Oxford) 2004;43:505–9. doi: 10.1093/rheumatology/keh087 14734787

[23] Montisci R , Vacca A, Garau P et al Detection of early impairment of coronary flow reserve in patients with systemic sclerosis. Ann Rheum Dis 2003;62:890–3. doi: 10.1136/ard.62.9.890 12922965 PMC1754674

[24] Gyllenhammar T , Kanski M, Engblom H et al Decreased global myocardial perfusion at adenosine stress as a potential new biomarker for microvascular disease in systemic sclerosis: a magnetic resonance study. BMC Cardiovasc Disord 2018;18:16. doi: 10.1186/s12872-018-0756-x 29382301 PMC5791343

[25] Kahaleh MB. Endothelin, an endothelial-dependent vasoconstrictor in scleroderma. Enhanced production and profibrotic action. Arthritis Rheum 1991;34:978–83. doi: 10.1002/art.1780340807 1859492

[26] Blake DR , Winyard P, Scott DG et al Endothelial cell cytotoxicity in inflammatory vascular diseases—the possible role of oxidised lipoproteins. Ann Rheum Dis 1985;44:176–82. doi: 10.1136/ard.44.3.176 3977420 PMC1001600

[27] Bruckdorfer KR , Hillary JB, Bunce T, Vancheeswaran R, Black CM. Increased susceptibility to oxidation of low-density lipoproteins isolated from patients with systemic sclerosis. Arthritis Rheum 1995;38:1060–7. doi: 10.1002/art.1780380807 7639801

[28] Worda M , Sgonc R, Dietrich H et al In vivo analysis of the apoptosis-inducing effect of anti-endothelial cell antibodies in systemic sclerosis by the chorionallantoic membrane assay. Arthritis Rheum 2003;48:2605–14. doi: 10.1002/art.11179 13130480

[29] Sprott H , Muller-Ladner U, Distler O et al Detection of activated complement complex C5b-9 and complement receptor C5a in skin biopsies of patients with systemic sclerosis (scleroderma). J Rheumatol 2000;27:402–4. 10685805

[30] Kahaleh MB , Fan PS. Mechanism of serum-mediated endothelial injury in scleroderma: identification of a granular enzyme in scleroderma skin and sera. Clin Immunol Immunopathol 1997;83:32–40. doi: 10.1006/clin.1996.4322 9073533

[31] De Luca G , Cavalli G, Campochiaro C et al Interleukin-1 and systemic sclerosis: getting to the heart of cardiac involvement. Front Immunol 2021;12:653950. doi: 10.3389/fimmu.2021.653950 33833766 PMC8021854

[32] Denton CP , Bickerstaff MC, Shiwen X et al Serial circulating adhesion molecule levels reflect disease severity in systemic sclerosis. Br J Rheumatol 1995;34:1048–54. doi: 10.1093/rheumatology/34.11.1048 8542206

[33] Springer TA. Adhesion receptors of the immune system. Nature 1990;346:425–34. doi: 10.1038/346425a0 1974032

[34] Rudnicka L , Majewski S, Blaszczyk M et al Adhesion of peripheral blood mononuclear cells to vascular endothelium in patients with systemic sclerosis (scleroderma). Arthritis Rheum 1992;35:771–5. doi: 10.1002/art.1780350710 1622415

[35] Stummvoll GH , Aringer M, Grisar J et al Increased transendothelial migration of scleroderma lymphocytes. Ann Rheum Dis 2004;63:569–74. doi: 10.1136/ard.2002.004838 15082489 PMC1754998

[36] Kobayashi S , Nagafuchi Y, Shoda H, Fujio K. The pathophysiological roles of regulatory T cells in the early phase of systemic sclerosis. Front Immunol 2022;13:900638. doi: 10.3389/fimmu.2022.900638 35686127 PMC9172592

[37] Reveille JD , Solomon DH; American College of Rheumatology Ad Hoc Committee of Immunologic Testing G. Evidence-based guidelines for the use of immunologic tests: anticentromere, Scl-70, and nucleolar antibodies. Arthritis Rheum 2003;49:399–412. doi: 10.1002/art.11113 12794797

[38] Chizzolini C , Raschi E, Rezzonico R et al Autoantibodies to fibroblasts induce a proadhesive and proinflammatory fibroblast phenotype in patients with systemic sclerosis. Arthritis Rheum 2002;46:1602–13. doi: 10.1002/art.10361 12115192

[39] Henault J , Robitaille G, Senecal JL, Raymond Y. DNA topoisomerase I binding to fibroblasts induces monocyte adhesion and activation in the presence of anti-topoisomerase I autoantibodies from systemic sclerosis patients. Arthritis Rheum 2006;54:963–73. doi: 10.1002/art.21646 16508979

[40] Baroni SS , Santillo M, Bevilacqua F et al Stimulatory autoantibodies to the PDGF receptor in systemic sclerosis. N Engl J Med 2006;354:2667–76. doi: 10.1056/NEJMoa052955 16790699

[41] Dinarello CA. Overview of the IL-1 family in innate inflammation and acquired immunity. Immunol Rev 2018;281:8–27. doi: 10.1111/imr.12621 29247995 PMC5756628

[42] Cavalli G , Colafrancesco S, Emmi G et al Interleukin 1alpha: a comprehensive review on the role of IL-1alpha in the pathogenesis and treatment of autoimmune and inflammatory diseases. Autoimmun Rev 2021;20:102763. doi: 10.1016/j.autrev.2021.102763 33482337

[43] Kawaguchi Y , Hara M, Wright TM. Endogenous IL-1alpha from systemic sclerosis fibroblasts induces IL-6 and PDGF-A. J Clin Invest 1999;103:1253–60. doi: 10.1172/JCI4304 10225968 PMC408350

[44] Guo H , Callaway JB, Ting JP. Inflammasomes: mechanism of action, role in disease, and therapeutics. Nat Med 2015;21:677–87. doi: 10.1038/nm.3893 26121197 PMC4519035

[45] Maleszewska M , Moonen JR, Huijkman N et al IL-1beta and TGFbeta2 synergistically induce endothelial to mesenchymal transition in an NFkappaB-dependent manner. Immunobiology 2013;218:443–54. doi: 10.1016/j.imbio.2012.05.026 22739237

[46] Zeisberg EM , Tarnavski O, Zeisberg M et al Endothelial-to-mesenchymal transition contributes to cardiac fibrosis. Nat Med 2007;13:952–61. doi: 10.1038/nm1613 17660828

[47] Martinez-Godinez MA , Cruz-Dominguez MP, Jara LJ et al Expression of NLRP3 inflammasome, cytokines and vascular mediators in the skin of systemic sclerosis patients. Isr Med Assoc J 2015;17:5–10. 25739168

[48] Artlett CM , Sassi-Gaha S, Hope JL, Feghali-Bostwick CA, Katsikis PD. Mir-155 is overexpressed in systemic sclerosis fibroblasts and is required for NLRP3 inflammasome-mediated collagen synthesis during fibrosis. Arthritis Res Ther 2017;19:144. doi: 10.1186/s13075-017-1331-z 28623945 PMC5473986

[49] Wei Y , Yan X, Yan L et al Inhibition of microRNA-155 ameliorates cardiac fibrosis in the process of angiotensin II-induced cardiac remodeling. Mol Med Rep 2017;16:7287–96. doi: 10.3892/mmr.2017.7584 28944921 PMC5865857

[50] Lane JR , Neumann DA, Lafond-Walker A, Herskowitz A, Rose NR. Role of IL-1 and tumor necrosis factor in coxsackie virus-induced autoimmune myocarditis. J Immunol 1993;151:1682–90. 8335952

[51] De Luca G , Cavalli G, Campochiaro C, Tresoldi M, Dagna L. Myocarditis: an interleukin-1-mediated disease? Front Immunol 2018;9:1335. doi: 10.3389/fimmu.2018.01335 29951067 PMC6008311

[52] Kumar A , Thota V, Dee L et al Tumor necrosis factor alpha and interleukin 1beta are responsible for in vitro myocardial cell depression induced by human septic shock serum. J Exp Med 1996;183:949–58. doi: 10.1084/jem.183.3.949 8642298 PMC2192364

[53] Van Tassell BW , Arena RA, Toldo S et al Enhanced interleukin-1 activity contributes to exercise intolerance in patients with systolic heart failure. PLoS One. 2012;7:e33438. doi: 10.1371/journal.pone.0033438 22438931 PMC3306393

[54] Baldeviano GC , Barin JG, Talor MV et al Interleukin-17A is dispensable for myocarditis but essential for the progression to dilated cardiomyopathy. Circ Res 2010;106:1646–55. doi: 10.1161/circresaha.109.213157 20378858

[55] Wei L , Abraham D, Ong V. The Yin and Yang of IL-17 in systemic sclerosis. Front Immunol 2022;13:885609. doi: 10.3389/fimmu.2022.885609 35603223 PMC9116143

[56] Di Battista M , Wasson CW, Alcacer-Pitarch B, Del Galdo F. Autonomic dysfunction in systemic sclerosis: a scoping review. Semin Arthritis Rheum 2023;63:152268. doi: 10.1016/j.semarthrit.2023.152268 37776665

[57] Nussinovitch U , Gendelman O, Rubin S et al Autonomic nervous system indices in patients with systemic sclerosis without overt cardiac disease. Isr Med Assoc J 2021;23:651–6. 34672448

[58] Masini F , Galiero R, Pafundi PC et al Autonomic nervous system dysfunction correlates with microvascular damage in systemic sclerosis patients. J Scleroderma Relat Disord 2021;6:256–63. doi: 10.1177/23971983211020617 35387218 PMC8922659

[59] Tadic M , Zlatanovic M, Cuspidi C et al The relationship between left ventricular deformation and heart rate variability in patients with systemic sclerosis: two- and three-dimensional strain analysis. Int J Cardiol 2017;236:145–50. doi: 10.1016/j.ijcard.2017.02.043 28222894

[60] Nihtyanova SI , Denton CP. Autoantibodies as predictive tools in systemic sclerosis. Nat Rev Rheumatol 2010;6:112–6. doi: 10.1038/nrrheum.2009.238 20125179

[61] Medsger TA Jr , Bombardieri S, Czirjak L et al Assessment of disease severity and prognosis. Clin Exp Rheumatol. 2003;21:S42-46. 12889222

[62] Steen VD , Medsger TA Jr. The palpable tendon friction rub: an important physical examination finding in patients with systemic sclerosis. Arthritis Rheum 1997;40:1146–51. doi: 10.1002/1529-0131(199706)40:6<1146::AID-ART19>3.0.CO;2-9 9182926

[63] Allanore Y , Meune C, Vonk MC; EUSTAR co-authors et al Prevalence and factors associated with left ventricular dysfunction in the EULAR Scleroderma Trial and Research group (EUSTAR) database of patients with systemic sclerosis. Ann Rheum Dis 2010;69:218–21. doi: 10.1136/ard.2008.103382 19279015

[64] Ali-Ahmed F , Dalgaard F, Al-Khatib SM. Sudden cardiac death in patients with myocarditis: evaluation, risk stratification, and management. Am Heart J 2020;220:29–40. doi: 10.1016/j.ahj.2019.08.007 31765933

[65] De Luca G , Campochiaro C, De Santis M et al Systemic sclerosis myocarditis has unique clinical, histological and prognostic features: a comparative histological analysis. Rheumatology (Oxford) 2020;59:2523–33. doi: 10.1093/rheumatology/kez658 31990340

[66] Lugrin J , Parapanov R, Milano G et al The systemic deletion of interleukin-1alpha reduces myocardial inflammation and attenuates ventricular remodeling in murine myocardial infarction. Sci Rep 2023;13:4006. doi: 10.1038/s41598-023-30662-4 36899010 PMC10006084

[67] Byers RJ , Marshall DA, Freemont AJ. Pericardial involvement in systemic sclerosis. Ann Rheum Dis 1997;56:393–4. doi: 10.1136/ard.56.6.393 9227172 PMC1752384

[68] Goldar G , Garraud C, Sifuentes AA et al Autoimmune pericarditis: multimodality imaging. Curr Cardiol Rep 2022;24:1633–45. doi: 10.1007/s11886-022-01785-3 36219367

[69] Welch TD. Constrictive pericarditis: diagnosis, management and clinical outcomes. Heart 2018;104:725–31. doi: 10.1136/heartjnl-2017-311683 29175978

[70] Smith JW , Clements PJ, Levisman J, Furst D, Ross M. Echocardiographic features of progressive systemic sclerosis (PSS). Correlation with hemodynamic and postmortem studies. Am J Med 1979;66:28–33. doi: 10.1016/0002-9343(79)90478-9 154294

[71] Meune C , Avouac J, Wahbi K et al Cardiac involvement in systemic sclerosis assessed by tissue-doppler echocardiography during routine care: a controlled study of 100 consecutive patients. Arthritis Rheum 2008;58:1803–9. doi: 10.1002/art.23463 18512815

[72] McWhorter J , LeRoy EC. Pericardial disease in scleroderma (systemic sclerosis). Am J Med 1974;57:566–75. doi: 10.1016/0002-9343(74)90008-4 4279550

[73] Hosoya H , Derk CT. Clinically symptomatic pericardial effusions in hospitalized systemic sclerosis patients: demographics and management. Biomed Res Int 2018;2018:6812082. doi: 10.1155/2018/6812082 29967777 PMC6008774

[74] Adler Y , Ristic AD, Imazio M et al Cardiac tamponade. Nat Rev Dis Primers 2023;9:36. doi: 10.1038/s41572-023-00446-1 37474539

[75] Fernandez Morales A , Iniesta N, Fernandez-Codina A et al Cardiac tamponade and severe pericardial effusion in systemic sclerosis: report of nine patients and review of the literature. Int J Rheum Dis 2017;20:1582–92. doi: 10.1111/1756-185X.12952 27943614

[76] Luo Y , Gordon JK, Xu J; PHAROS Investigators et al Prognostic significance of pericardial effusion in systemic sclerosis-associated pulmonary hypertension: analysis from the PHAROS Registry. Rheumatology (Oxford) 2024;63:1251–8. doi: 10.1093/rheumatology/kead368 37478347 PMC11065440

[77] Ross L , Costello B, Brown Z et al Myocardial fibrosis and arrhythmic burden in systemic sclerosis. Rheumatology (Oxford) 2022;61:4497–502. doi: 10.1093/rheumatology/keac065 35136975 PMC9629381

[78] Fairley JL , Ross L, Quinlivan A et al Sudden cardiac death, arrhythmias and abnormal electrocardiography in systemic sclerosis: a systematic review and meta-analysis. Semin Arthritis Rheum 2023;62:152229. doi: 10.1016/j.semarthrit.2023.152229 37354723

[79] Wong CX , Brown A, Lau DH et al Epidemiology of sudden cardiac death: global and regional perspectives. Heart Lung Circ 2019;28:6–14. doi: 10.1016/j.hlc.2018.08.026 30482683

[80] Tzelepis GE , Kelekis NL, Plastiras SC et al Pattern and distribution of myocardial fibrosis in systemic sclerosis: a delayed enhanced magnetic resonance imaging study. Arthritis Rheum 2007;56:3827–36. doi: 10.1002/art.22971 17968945

[81] Batani V , Palmisano A, Campochiaro C et al The complex diagnosis of primary heart involvement in systemic sclerosis: red flags to drive the choice of cardiac magnetic resonance. Rheumatology (Oxford) 2025;64:5004–13. doi: 10.1093/rheumatology/keaf234 40353849

[82] Bissell L-A , Dumitru RB, Erhayiem B et al Incidental significant arrhythmia in scleroderma associates with cardiac magnetic resonance measure of fibrosis and hs-TnI and NT-proBNP. Rheumatology (Oxford) 2019;58:1221–6. doi: 10.1093/rheumatology/key430 30690570 PMC6587914

[83] Terrier B , Dechartres A, Gouya H et al Cardiac intravoxel incoherent motion diffusion-weighted magnetic resonance imaging with T1 mapping to assess myocardial perfusion and fibrosis in systemic sclerosis: association with cardiac events from a prospective cohort study. Arthritis Rheumatol 2020;72:1571–80. doi: 10.1002/art.41308 32379399

[84] De Luca G , Bosello SL, Gabrielli FA et al Prognostic role of ventricular ectopic beats in systemic sclerosis: a prospective cohort study shows ECG indexes predicting the worse outcome. PLoS One. 2016;11:e0153012. doi: 10.1371/journal.pone.0153012 27101136 PMC4839708

[85] Hinchcliff M , Desai CS, Varga J, Shah SJ. Prevalence, prognosis, and factors associated with left ventricular diastolic dysfunction in systemic sclerosis. Clin Exp Rheumatol. 2012;30:S30-37. 22338601 PMC3507505

[86] Hinze AM , Perin J, Woods A et al Diastolic dysfunction in systemic sclerosis: risk factors and impact on mortality. Arthritis Rheumatol 2022;74:849–59. doi: 10.1002/art.42054 34927390 PMC9050815

[87] Giuca A , Gegenava T, Mihai CM et al Sclerodermic cardiomyopathy-a state-of-the-art review. Diagnostics (Basel) 2022;12:669. doi: 10.3390/diagnostics12030669 PMC8947572 35328222

[88] Pieroni M , De Santis M, Zizzo G et al Recognizing and treating myocarditis in recent-onset systemic sclerosis heart disease: potential utility of immunosuppressive therapy in cardiac damage progression. Semin Arthritis Rheum 2014;43:526–35. doi: 10.1016/j.semarthrit.2013.07.006 23932313

[89] Feher A , Miller EJ, Peters DC et al Impaired left-ventricular global longitudinal strain by feature-tracking cardiac MRI predicts mortality in systemic sclerosis. Rheumatol Int 2023;43:849–58. doi: 10.1007/s00296-023-05294-6 36894756 PMC12073471

[90] Knight DS , Karia N, Cole AR et al Distinct cardiovascular phenotypes are associated with prognosis in systemic sclerosis: a cardiovascular magnetic resonance study. Eur Heart J Cardiovasc Imaging 2023;24:463–71. doi: 10.1093/ehjci/jeac120 35775814 PMC10029850

[91] Schattke S , Knebel F, Grohmann A et al Early right ventricular systolic dysfunction in patients with systemic sclerosis without pulmonary hypertension: a Doppler Tissue and Speckle Tracking echocardiography study. Cardiovasc Ultrasound 2010;8:3. doi: 10.1186/1476-7120-8-3 20096122 PMC2822748

[92] Huez S , Roufosse F, Vachiery JL et al Isolated right ventricular dysfunction in systemic sclerosis: latent pulmonary hypertension? Eur Respir J 2007;30:928–36. doi: 10.1183/09031936.00025607 17690126

[93] Hsu S , Kokkonen-Simon KM, Kirk JA et al Right ventricular myofilament functional differences in humans with systemic sclerosis-associated versus idiopathic pulmonary arterial hypertension. Circulation 2018;137:2360–70. doi: 10.1161/CIRCULATIONAHA.117.033147 29352073 PMC5976528

[94] Hesselstrand R , Wildt M, Ekmehag B, Wuttge DM, Scheja A. Survival in patients with pulmonary arterial hypertension associated with systemic sclerosis from a Swedish single centre: prognosis still poor and prediction difficult. Scand J Rheumatol 2011;40:127–32. doi: 10.3109/03009742.2010.508751 20858146

[95] Lefevre G , Dauchet L, Hachulla E et al Survival and prognostic factors in systemic sclerosis-associated pulmonary hypertension: a systematic review and meta-analysis. Arthritis Rheum 2013;65:2412–23. doi: 10.1002/art.38029 23740572

[96] Bruera S , Sidanmat H, Molony DA et al Stem cell transplantation for systemic sclerosis. Cochrane Database Syst Rev. 2022;7:CD011819. doi: 10.1002/14651858.CD011819.pub2 35904231 PMC9336163

[97] Fujimaki K , Maruta A, Yoshida M et al Severe cardiac toxicity in hematological stem cell transplantation: predictive value of reduced left ventricular ejection fraction. Bone Marrow Transplant 2001;27:307–10. doi: 10.1038/sj.bmt.1702783 11277179

[98] Sullivan KM , Goldmuntz EA, Keyes-Elstein L; SCOT Study Investigators et al Myeloablative autologous stem-cell transplantation for severe scleroderma. N Engl J Med 2018;378:35–47. doi: 10.1056/nejmoa1703327 29298160 PMC5846574

[99] Markousis-Mavrogenis G , Bournia VK, Sfikakis PP, Mavrogeni SI. Raynaud phenomenon and microvasculopathy in systemic sclerosis: multi-modality imaging for diagnosis and evaluation. Curr Opin Rheumatol 2023;35:324–33. doi: 10.1097/BOR.0000000000000965 37582056

[100] Marinescu MA , Loffler AI, Ouellette M et al Coronary microvascular dysfunction, microvascular angina, and treatment strategies. JACC Cardiovasc Imaging 2015;8:210–20. doi: 10.1016/j.jcmg.2014.12.008 25677893 PMC4384521

[101] Alexander EL , Firestein GS, Weiss JL et al Reversible cold-induced abnormalities in myocardial perfusion and function in systemic sclerosis. Ann Intern Med 1986;105:661–8. doi: 10.7326/0003-4819-105-5-661 3767147

[102] Gustafsson R , Mannting F, Kazzam E, Waldenström A, Hällgren R. Cold-induced reversible myocardial ischaemia in systemic sclerosis. Lancet 1989;2:475–9. doi: 10.1016/s0140-6736(89)92088-6 2570187

[103] Mizuno R , Fujimoto S, Saito Y, Nakamura S. Cardiac Raynaud’s phenomenon induced by cold provocation as a predictor of long-term left ventricular dysfunction and remodelling in systemic sclerosis: 7-year follow-up study. Eur J Heart Fail 2010;12:268–75. doi: 10.1093/eurjhf/hfp198 20071354

[104] Akram MR , Handler CE, Williams M et al Angiographically proven coronary artery disease in scleroderma. Rheumatology (Oxford) 2006;45:1395–8. doi: 10.1093/rheumatology/kel120 16606654

[105] Di Battista M , Barsotti S, Della Rossa A, Mosca M. Cardiovascular burden in systemic sclerosis: QRISK3 versus Framingham for risk estimation. Mod Rheumatol 2022;32:584–8. doi: 10.1093/mr/roab011 34888692

[106] Risks C ; Global Burden of Cardiovascular D. Global, regional, and national burden of cardiovascular diseases and risk factors in 204 countries and territories, 1990-2023. J Am Coll Cardiol 2025;86:2167–243. doi: 10.1016/j.jacc.2025.08.015 40990886

[107] Bissell L-A , Anderson M, Burgess M et al Consensus best practice pathway of the UK Systemic Sclerosis Study group: management of cardiac disease in systemic sclerosis. Rheumatology (Oxford) 2017;56:912–21. doi: 10.1093/rheumatology/kew488 28160468

[108] De Luca G , Matucci-Cerinic M, Mavrogeni SI. Diagnosis and management of primary heart involvement in systemic sclerosis. Curr Opin Rheumatol 2024;36:76–93. doi: 10.1097/BOR.0000000000000990 37962165

[109] Wang G , Yang Q, Wu S et al Molecular imaging of fibroblast activity in pressure overload heart failure using [(68) Ga]Ga-FAPI-04 PET/CT. Eur J Nucl Med Mol Imaging 2023;50:465–74. doi: 10.1007/s00259-022-05984-6 36171409

[110] Ferreira VM , Schulz-Menger J, Holmvang G et al Cardiovascular magnetic resonance in nonischemic myocardial inflammation: expert recommendations. J Am Coll Cardiol 2018;72:3158–76. doi: 10.1016/j.jacc.2018.09.072 30545455

[111] Mueller KAL , Mueller II, Eppler David, et al Clinical and histopathological features of patients with systemic sclerosis undergoing endomyocardial biopsy. PLoS One. 2015;10:e0126707. doi: 10.1371/journal.pone.0126707 25966025 PMC4428754

[112] Batani V , Dagna L, De Luca G. Therapeutic strategies for primary heart involvement in systemic sclerosis. Rheumatol Immunol Res 2024;5:72–82. doi: 10.1515/rir-2024-0010 39015843 PMC11248560

[113] Panopoulos S , Mavrogeni S, Vlachopoulos C, Sfikakis PP. Cardiac magnetic resonance imaging before and after therapeutic interventions for systemic sclerosis-associated myocarditis. Rheumatology 2023;62:1535–42. doi: 10.1093/rheumatology/keac504 36083014

[114] De Luca G , De Santis M, Batani V et al Immunosuppressive therapy to treat newly diagnosed primary heart involvement in patients with systemic sclerosis: An Italian cardiac magnetic resonance based study. Semin Arthritis Rheum 2025;71:152622. doi: 10.1016/j.semarthrit.2024.152622 39826307

[115] Mavrogeni S , Koutsogeorgopoulou L, Karabela G et al Silent myocarditis in systemic sclerosis detected by cardiovascular magnetic resonance using Lake Louise criteria. BMC Cardiovasc Disord 2017;17:187. doi: 10.1186/s12872-017-0619-x 28716007 PMC5513128

[116] Campochiaro C , De Luca G, Tomelleri A et al Tocilizumab for the treatment of myocardial inflammation shown by cardiac magnetic resonance: report of two cases and rationale for its therapeutic use. J Clin Rheumatol. 2021;27:S476–9. doi: 10.1097/RHU.0000000000001194 31790000

[117] Mantero JC , Kishore N, Ziemek J et al Randomised, double-blind, placebo-controlled trial of IL1-trap, rilonacept, in systemic sclerosis. A phase I/II biomarker trial. Clin Exp Rheumatol 2018;36(Suppl 113):146–9. 30277862

[118] Adler Y , Charron P, Imazio M; ESC Scientific Document Group et al 2015 ESC Guidelines for the diagnosis and management of pericardial diseases: the Task Force for the Diagnosis and Management of Pericardial Diseases of the European Society of Cardiology (ESC)Endorsed by: the European Association for Cardio-Thoracic Surgery (EACTS). Eur Heart J 2015;36:2921–64. doi: 10.1093/eurheartj/ehv318 26320112 PMC7539677

[119] Goswami RP , Ray A, Chatterjee M et al Rituximab in the treatment of systemic sclerosis-related interstitial lung disease: a systematic review and meta-analysis. Rheumatology (Oxford) 2021;60:557–67. doi: 10.1093/rheumatology/keaa550 33164098

[120] Cole A , Ong VH, Denton CP. Renal disease and systemic sclerosis: an update on scleroderma renal crisis. Clin Rev Allergy Immunol 2023;64:378–91. doi: 10.1007/s12016-022-08945-x 35648373 PMC10167155

[121] Kontzias A , Barkhodari A, Yao Q. Pericarditis in systemic rheumatologic diseases. Curr Cardiol Rep 2020;22:142. doi: 10.1007/s11886-020-01415-w 32910306

[122] Hemnes AR , Gaine SP, Wiener CM. Poor outcomes associated with drainage of pericardial effusions in patients with pulmonary arterial hypertension. South Med J 2008;101:490–4. doi: 10.1097/SMJ.0b013e31816c0169 18414173

[123] Andreis A , Imazio M, Casula M, Avondo S, Brucato A. Recurrent pericarditis: an update on diagnosis and management. Intern Emerg Med 2021;16:551–8. doi: 10.1007/s11739-021-02639-6 33641044 PMC7914388

[124] Imazio M , Brucato A, Mayosi BM et al Medical therapy of pericardial diseases: part I: idiopathic and infectious pericarditis. J Cardiovasc Med (Hagerstown) 2010;11:712–22. doi: 10.2459/JCM.0b013e3283340b97 20736783

[125] Haley JH , Tajik AJ, Danielson GK et al Transient constrictive pericarditis: causes and natural history. J Am Coll Cardiol 2004;43:271–5. doi: 10.1016/j.jacc.2003.08.032 14736448

[126] Glikson M , Nielsen JC, Kronborg MB; ESC Scientific Document Group et al 2021 ESC Guidelines on cardiac pacing and cardiac resynchronization therapy: developed by the Task Force on cardiac pacing and cardiac resynchronization therapy of the European Society of Cardiology (ESC) With the special contribution of the European Heart Rhythm Association (EHRA). Rev Esp Cardiol (Engl Ed) 2022;75:430. doi: 10.1016/j.rec.2022.04.004 35525571

[127] Brugada J , Katritsis DG, Arbelo E; ESC Scientific Document Group et al 2019 ESC Guidelines for the management of patients with supraventricular tachycardiaThe Task Force for the management of patients with supraventricular tachycardia of the European Society of Cardiology (ESC). Eur Heart J 2020;41:655–720. doi: 10.1093/eurheartj/ehz467 31504425

[128] Zeppenfeld K , Tfelt-Hansen J, de Riva M; ESC Scientific Document Group et al 2022 ESC Guidelines for the management of patients with ventricular arrhythmias and the prevention of sudden cardiac death. Eur Heart J 2022;43:3997–4126. doi: 10.1093/eurheartj/ehac262 36017572

[129] Hindricks G , Potpara T, Dagres N; ESC Scientific Document Group et al 2020 ESC Guidelines for the diagnosis and management of atrial fibrillation developed in collaboration with the European Association for Cardio-Thoracic Surgery (EACTS): the Task Force for the diagnosis and management of atrial fibrillation of the European Society of Cardiology (ESC) Developed with the special contribution of the European Heart Rhythm Association (EHRA) of the ESC. Eur Heart J 2021;42:373–498. doi: 10.1093/eurheartj/ehaa612 32860505

[130] Valentini G , Huscher D, Riccardi A et al Vasodilators and low-dose acetylsalicylic acid are associated with a lower incidence of distinct primary myocardial disease manifestations in systemic sclerosis: results of the DeSScipher inception cohort study. Ann Rheum Dis 2019;78:1576–82. doi: 10.1136/annrheumdis-2019-215486 31391176

[131] McDonagh TA , Metra M, Adamo M; ESC Scientific Document Group et al 2021 ESC Guidelines for the diagnosis and treatment of acute and chronic heart failure. Eur Heart J 2021;42:3599–726. doi: 10.1093/eurheartj/ehab368 34447992

[132] McDonagh TA , Metra M, Adamo M; ESC Scientific Document Group et al 2023 Focused Update of the 2021 ESC Guidelines for the diagnosis and treatment of acute and chronic heart failure. Eur Heart J 2023;44:3627–39. doi: 10.1093/eurheartj/ehad195 37622666

[133] Solomon SD , McMurray JJV, Vaduganathan M; FINEARTS-HF Committees and Investigators et al Finerenone in heart failure with mildly reduced or preserved ejection fraction. N Engl J Med 2024;391:1475–85. doi: 10.1056/NEJMoa2407107 39225278

[134] Wind S , Schmid U, Freiwald M et al Clinical pharmacokinetics and pharmacodynamics of nintedanib. Clin Pharmacokinet 2019;58:1131–47. doi: 10.1007/s40262-019-00766-0 31016670 PMC6719436

[135] Liakouli V , Ciancio A, Del Galdo F, Giacomelli R, Ciccia F. Systemic sclerosis interstitial lung disease: unmet needs and potential solutions. Nat Rev Rheumatol 2024;20:21–32. doi: 10.1038/s41584-023-01044-x 37923862

[136] Ninagawa K , Kato M, Tsuneta S et al Beneficial effects of nintedanib on cardiomyopathy in patients with systemic sclerosis: a pilot study. Rheumatology (Oxford) 2023;62:2550–5. doi: 10.1093/rheumatology/keac674 36458921

[137] Cattaneo M , Porretta AP, Gallino A. Ranolazine: drug overview and possible role in primary microvascular angina management. Int J Cardiol 2015;181:376–81. doi: 10.1016/j.ijcard.2014.12.055 25555283

[138] Vignaux O , Allanore Y, Meune C et al Evaluation of the effect of nifedipine upon myocardial perfusion and contractility using cardiac magnetic resonance imaging and tissue Doppler echocardiography in systemic sclerosis. Ann Rheum Dis 2005;64:1268–73. doi: 10.1136/ard.2004.031484 15708883 PMC1755644

[139] Csiki Z , Garai I, Shemirani AH et al The effect of metoprolol alone and combined metoprolol-felodipin on the digital microcirculation of patients with primary Raynaud’s syndrome. Microvasc Res 2011;82:84–7. doi: 10.1016/j.mvr.2011.04.004 21515290

[140] Godfraind T. Calcium channel blockers in cardiovascular pharmacotherapy. J Cardiovasc Pharmacol Ther 2014;19:501–15. doi: 10.1177/1074248414530508 24872348

